# New Perspectives about Drug Candidates Targeting HTLV-1 and Related Diseases

**DOI:** 10.3390/ph16111546

**Published:** 2023-11-02

**Authors:** Milena Cristina Martins da Silva, Renan Stefferson Barradas Pereira, Antonia Cherlly Aparecida Araujo, Ednilson Gregorio da Silva Filho, Anderson de Lima Dias, Kassio Silva Cavalcante, Maísa Silva de Sousa

**Affiliations:** 1 Tropical Medicine Center, Federal University of Para, Belem 66055-240, Brazil; 2 Institute of Health Sciences, Faculty of Pharmacy, Federal University of Para, Belem 66079-420, Brazil

**Keywords:** HTLV-1-related diseases, drug treatment, ATLL, HAM/TSP, molecular docking

## Abstract

Among the human T-lymphotropic virus (HTLV) types, HTLV-1 is the most prevalent, and it has been linked to a spectrum of diseases, including HAM/TSP, ATLL, and hyperinfection syndrome or disseminated strongyloidiasis. There is currently no globally standard first-line treatment for HTLV-1 infection and its related diseases. To address this, a comprehensive review was conducted, analyzing 30 recent papers from databases PubMed, CAPES journals, and the Virtual Health Library (VHL). The studies encompassed a wide range of therapeutic approaches, including antiretrovirals, immunomodulators, antineoplastics, amino acids, antiparasitics, and even natural products and plant extracts. Notably, the category with the highest number of articles was related to drugs for the treatment of ATLL. Studies employing mogamulizumab as a new perspective for ATLL received greater attention in the last 5 years, demonstrating efficacy, safe use in the elderly, significant antitumor activity, and increased survival time for refractory patients. Concerning HAM/TSP, despite corticosteroid being recommended, a more randomized clinical trial is needed to support treatment other than corticoids. The study also included a comprehensive review of the drugs used to treat disseminated strongyloidiasis in co-infection with HTLV-1, including their administration form, in order to emphasize gaps and facilitate the development of other studies aiming at better-directed methodologies. Additionally, docking molecules and computer simulations show promise in identifying novel therapeutic targets and repurposing existing drugs. These advances are crucial in developing more effective and targeted treatments against HTLV-1 and its related diseases.

## 1. Introduction

The human T-lymphotropic virus (HTLV) belonging to the *Retroviridae* family was discovered and isolated for the first time in 1980, and since then [1], it has been the subject of scientific discussions due to its relationship with other infections and clinical conditions since it has the capacity to infect the cells of the immune system, reducing the body’s defense [2].

Currently, four types of HTLV are known: 1, 2, 3, and 4. Among these, types 1 and 2 are the most studied because they cause the highest number of infections globally [3]. HTLV-1 affects approximately 15 to 20 million individuals, with the highest rates coming from Japan, sub-Saharan Africa, South America, and the Caribbean. It affects both men and women, with a higher seroprevalence in people over 50 years of age, mainly females.

HTLV-2 affects about 800,000 individuals, with the majority in the Americas, mainly in the United States (between 400 and 500 thousand) and Brazil (between 200 and 250 thousand), but it can also be found in Europe and Central Africa. HTLV-2 is often detected in Native American populations and among IV drug users [3,4].

Types 1 and 2 share characteristics: both rely on cell–cell contact for transmission and use envelope glycoproteins to mediate attachment and entry into other cells. In addition, they use neuropilin-1 (NRP1) and glucose transporter 1 (GLUT1) receptors to bind and enter cells, respectively. However, they differ in the actions of regulatory proteins Tax-1 (HTLV-1) and Tax-2 (HTLV-2) and products of antisense genes HBZ (type 1) and APH-2 (type 2), leading to different clinical manifestations. Another difference observed is regarding cell tropism; HTLV-1 has tropism for CD4+ T cells, while type 2 has tropism for CD8+ T cells. Most HTLV-2 carriers are asymptomatic, but some neurological conditions such as HTLV-1-associated myelopathy/tropical spastic paraparesis (HAM/TSP) may occur [3,4,5].

The discovery of HTLV-3 was reported by researchers in 2005 in two asymptomatic individuals living in the rainforest area of South Cameroon [6,7]. More recently, the same teams reported the discovery of two additional HTLV-3 strains in other individuals from Cameroon [8,9]. The fourth HTLV type (HTLV-4) consists of a unique human strain found in the PBMCs obtained from a hunter living in Cameroon [7].

Among the types of HTLV, the most frequent is type 1 [4,5,6,7,8,9,10]. The transmission occurs mostly through breastfeeding in infancy or sexual intercourse in adults, or through contaminated blood products and tissue transplantation [11]. Although most HTLV-1 carriers remain asymptomatic for life, part of them will develop related diseases. Several serious diseases are thought to be caused by or strongly associated with the virus. These diseases show no specific symptoms; therefore, unless HTLV-1 is considered first and serology ordered, the correct diagnosis is not possible [2].

The main diseases related to HTLV-1 infection include HAM/TSP, adult T-cell leukaemia/lymphoma (ATLL), and hyperinfection syndrome or disseminated strongyloidiasis. However, other conditions such as HTLV-1-associated uveitis (HAU), infective dermatitis, bronchiectasis, bronchitis and bronchiolitis, seborrheic dermatitis, Sjögren’s syndrome, rheumatoid arthritis, fibromyalgia, and ulcerative colitis also have been related to this virus infection [12]. 

Although the mechanism of HTLV-1 pathogenesis is not fully understood, it is known that the virus infects dendritic cells, macrophages, monocytes, CD8+ T lymphocytes, and mainly CD4+ T lymphocytes [13]. The mechanisms of HTLV-1 interaction with the host, host responses, and its immunogenetic characteristics seem to be the most important variables for the pathogenesis of related diseases. Nevertheless, it is still unknown why some people develop severe cases of HAM or ATLL, others have moderate disease, and many others are asymptomatic [14].

The management of patients living with HTLV-1, as well as the treatment of diseases associated with the virus, remains a challenge worldwide. Although studies about the virus and associated diseases treatment have been developed since the 1990s [15,16], some therapeutic agents used to treat its associated disease, such as ATLL, are not universally available. Zidovudine and interferon-alpha (AZT/IFN) have been used to treat ATLL in different parts of the world, including countries in Europe and America. In Japan, where several studies about the theme have been conducted [17], regimens other than AZT/IFN are used to treat ATLL. Mogamulizumab and certain components of the vincristine, cyclophosphamide, doxorubicin, and prednisone (VCAP); doxorubicin, ranimustine, and prednisone (AMP); and vindesine, etoposide, carboplatin, and prednisone (VECP) chemotherapy regimen (modified LSG15) are combined to treat ATLL in this country, but some of these drugs are unavailable in other countries, resulting in different treatment strategies around the world [18].

Despite experts mostly agreeing treatments listed are appropriate, the International Consensus report review for ATLL has the objective of benefiting patient care through recommended good practice by consensus. It particularly highlights the following points: the mechanism of action of some drugs is not completely understood; there are patients who are resistant to therapy (e.g., with AZT/IFN); several advances in the clinical management of patients with ATLL, particularly in Japan with the use of drugs such as lenalidomide, have been made; and for ensuring the consensus is continually updated to establish evidence-based practice guidelines, more studies, including clinical trials, are needed [18].

The international consensus guidelines for the management of HAM, an important HTLV-1 associated disease, describes that pharmacological treatment aims to modify the disease progression, reducing symptoms and increasing mobility. Despite there being evidence to support the use of corticoids methylprednisolone and prednisolone for progressive disease dependent on the disease stage and patients’ condition, the evidence base for guiding treatment for patients with HAM/TSP is extremely limited, mainly for therapies other than corticoids, and according to this consensus, higher-quality evidence to support any recommendations is an urgent need because the potential effect of the current (and future) therapies is uncertain [19].

In the absence of an effective vaccine to prevent the infection, drugs are the main tool for the treatment of patients. Antiretroviral therapy does not eliminate viral reservoirs, but it can disrupt the virus life cycle and reduce the replication. Over the years, viral targets for anti-HTLV-1 drugs have been studied. The regulatory proteins encoded by proviral DNA, such as Tax and HBZ proteins, that possibly act through the induction of cell growth, have been pointed out as possible targets [20], but the World Health Organization (WHO) has not produced any guidelines or recommendations on managing HTLV-1 infection or HTLV-1-related conditions yet because scientific findings are still controversial [14]. In this context, therapeutic options and the lack of standard first-line treatments represent a challenge for the medical community. The fact that existing treatments are heterogeneous among endemic countries makes this issue even more challenging. The knowledge about better targeted and more effective new drugs is essential to address this threat to global health. This review aimed to gather and discuss the information developed in the last five years about this topic.

## 2. Materials and Methods

**Figure 1:**
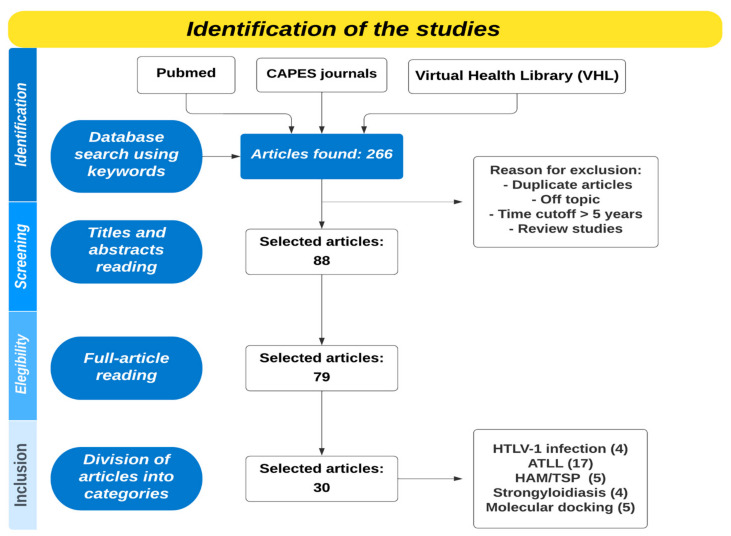
Article selection flowchart.

For this review, the databases PubMed, CAPES journals, and the Virtual Health Library (VHL) were examined using the following phrases: (Drug) AND (HTLV-1). The search was made between April and May 2023, and only articles from the last five years in Portuguese or English were accepted. The selection was made by the authors together: firstly, by reading titles and abstracts, then by reading the full article according to the flowchart (Figure 1). In addition, to facilitate the organization and tabulation of the data, a table with the title, authors, and treatments were made. From the complete reading of the articles, only 30 were relevant to the proposed theme. These articles were included and analyzed according to the categories described below: treatment for HTLV-1 infection, drugs to treat ATLL, drugs to treat HAM/TSP, and treatment of Strongyloidiasis and molecular docking as a tool to find innovative treatments. These categories were determined according to the articles found as a result of the search in databases. 

### 2.1. New Studies about Drugs to Treat HTLV-1 Infection

The mechanism of HTLV-1 pathogenesis is still not fully understood. Among all the regulatory proteins encoded by proviral DNA, the Tax and HBZ proteins seem to be essential for viral pathogenesis, possibly through the induction of cell growth [21]. For this reason, they are often studied as a target for drugs. However, due to the lack of complete elucidation of the defense mechanism, the treatment is still uncertain, and studies have been developed to discover new drugs and/or determine which would be the most appropriate to be used singly or in combination as the first choice to treat patients with HTLV-1.

In this context, the potential of 1,2,3-triazole derivatives, obtained and purified by flash chromatography, was assessed by cell-based assay using the resazurin reduction method and evaluated towards cell cycle, as well as in terms of apoptosis and Tax/GFP expression analyses through flow cytometry. From the screening of 25 compounds, three active and non-cytotoxic compounds were found. They could decrease the metabolic activity of the HTLV-1-infected cell line (MT-2), promoting the significant activation of effector caspase-3/7 compared to the control, and/or interfering in LTR transactivation and/or Tax expression (Table 1). The compounds can potentially contribute as a drug for ATLL patients [22].

Other synthetic compounds tested were the ones belonging to mesoionic class. The (*E*)-3-phenyl-5-(phenylamino)-2-styryl-1,3,4-thiadiazol-3-iuchloride derivatives were evaluated against MT-2 and C92 cell lines infected with human HTLV-1 and non-infected cells (Jurkat). The compounds also had their cytotoxicity assessed using 3-(4,5-dimethylthiazol-2-yl)-2,5-diphenyltetrazolium bromide (MTT) assays. The results showed IC_50_ values of all compounds in the range of 1.51–7.70 μM in HTLV-1-infected and non-infected cells. One of the compounds induced necrosis after 24 h in Jurkat and MT-2 cell lines. The experimental (fluorimetric method) and theoretical (molecular docking) results suggested that the mechanism of action could be related to its capacity to intercalate into DNA, and they presented spontaneous and moderate interaction with albumin in human serum albumin (HSA)-binding affinity assay by multiple spectroscopic techniques (circular dichroism, steady-state and time-resolved fluorescence), zeta potential, and molecular docking calculations, indicating good biodistribution in the human bloodstream (Table 1) [23].

A drug that has been studied is pomalidomide (pom). A group of researchers who had already tested this drug in vitro and proved it increased the susceptibility of HTLV-1-infected cells to NK and CTL killing used the rhesus macaque model to determine if pom treatment of infected individuals activates the host immune system and allows for the recognition and clearance of HTLV-1-infected cells. The results suggested pomalidomide can enhance the immune response to HTLV-1 infection, but this response is not maintained, and the authors suggested a combination with other well-tolerated drugs such as lenalidomide (Table 1) [24].

Considering the emergence of drug-resistant viruses, as well as the low efficacy, high cost, toxicity, and low bioavailability of the current antiviral drugs, returning to traditional herbal medicine is considered an alternative approach. Several medicinal plants and natural products have been identified as possible alternatives to fight HTLV-1 infection and its related diseases. For example, curcumin and its analogues have shown significant effects against retroviruses [25]. *Bidens pilosa* extracts have demonstrated growth suppressive effects on HTLV-1-infected T-cell lines and ATLL cells [26]. Green tea catechins, especially epigallocatechin-3-gallate (EGCG), have shown antiviral effects against HTLV-1, achieved by suppressing HTLV-I pX and Tax gene expression [27].

**Table 1:** New drugs, molecules, or natural products for HTLV-1 infection treatment.

HTLV-1 Infection				
Drug/Molecule/ Natural Product	Activity	Study Methodology	Year	Author
1,2,3-Triazole tethered fused heterocyclic ring derivatives	Compounds induced S-phase cell cycle arrest; promoted apoptosis; and reduced GFP expression in an inducible-Tax reporter cell, which suggests an effect on Tax.	Cell-based assay using resazurin reduction method, and evaluation towards cell cycle, apoptosis and Tax/GFP expression analyzes through flow cytometry.	2020	[22]
Alcoholic extract from *Eucalyptus camaldulensis*	Inhibited Tax induced activation of NF-κB, SRF-dependent promoters, and HTLV-1 LTR.	Evaluated the activity of the extract by testing its influence on Tax-induced activity of NF-κB and HTLV-1 LTR in Jurkat cells.	2020	[28]
(*E*)-3-Phenyl-5-(phenylamino)-2-styryl-1,3,4-thiadiazol-3-ium chloride derivatives	Caused necrosis of Jurkat and MT-2 cells infected with HTLV-1 after 24 h, maybe due to its capacity to intercalate into DNA	Biological evaluation against MT-2 and C92 cell lines infected with human T-cell lymphotropic virus type-1 (HTLV-1)	2020	[23]
Pomalidomide (pom)	Can enhance the immune response to HTLV-1 infection, but this response is not maintained.	Rhesus macaque model. The pom (0.2 mg/kg) was administered orally to four HTLV-1-infected macaques over a 24-day period and collected blood, urine, and bone marrow samples.	2022	[24]

NF-ĸB: nuclear factor-kappa B; pom: pomalidomide.

Knowing that many drugs were discovered and extracted from plant material, plants can be an excellent resource for the discovery of new treatments. The *Eucalyptus camaldulensis* (Ec) alcoholic extract was evaluated for this purpose by testing its influence on the Tax-induced activity of NF-ĸB and HTLV-1 LTR in Jurkat cells. The results showed that *Ec* inhibited Tax-induced activation of NF-ĸB, SRF-dependent promoters, and HTLV-1 LTR. The 40%-MeOH fraction of this extract, rich with polyphenols, offered the highest inhibitory effect against Tax activities, but further studies are required to isolate the active component/s in this extract (Table 1) [28].

A new perspective is the development of vaccines based on peptides, an alternative that can contain different epitopes in just one dose. One study, observing the activation of CD8+ T lymphocytes and the majority presence (88%) of the HLA-A2 and A24 alleles in HTLV-I infected individuals, conducted an immunoproteomic analysis to identify MHC-I-restricted epitopes capable of binding to these alleles and eliciting an anti-HTLV-I response by polyclonal T cells. Using the MT-2 and SLB-1 cell lines (HLA-A2+ and A24+), 6 epitopes were identified (IIN, ITN, PLL, FTD, FLN, and LFA), all originating from proteins whose actions are essential for HTLV-I pathogenesis. In vitro assays showed that CD8+ T cells activated by these epitopes secrete cytokines with cytotoxic and antiviral activity (such as IFN-γ and TNF-α). In in vivo assays, immunogenicity was confirmed by ELISpot, CD107 degranulation assay, and MagPix MILLIPLEX analysis [29].

### 2.2. Updates in Drug Research for the Treatment of Adult T-Cell Leukemia/Lymphoma (ATLL)

The adult T-cell leukemia/lymphoma (ATLL) is an aggressive lymphoproliferative disease of mature T cells, etiologically related with the human T-lymphotropic virus type 1 (HTLV-1). It has various clinical features, as well as risk factors such as maternal transmission, older age, increased proviral load in peripheral blood, and family history of ATLL that have already been identified [4,5,6,7,8,9,10,11,12,13,14,15,16,17,18,19,20,21,22,23,24,25,26,27,28,29,30]. 

Although the clinical evolution of the types of ATLL is different, the more aggressive forms have an unfavorable prognosis resulting in very short overall survival (OS) [14]. In recent years, many drugs have been tested against ATLL to develop a more effective therapeutic regimen; however, there is no worldwide standard use of drugs to treat ATLL due to the heterogeneity of available drugs in countries with high prevalence of the disease and scarcity of clinical trials that investigate these drugs among first-line treatment options. The international consensus regarding the treatment of ATLL uses GRADE criteria for the quality of evidence and states that the level of evidence for ATLL should be regarded as low or very low, equivalent to a GRADE evidence score of C or D [18].

In Japan, one of the areas with the highest occurrence of ATLL, chemotherapy is used with the association of several drugs such as Hiper-CVAD (cyclophosphamide, vincristine, doxorubicin, and dexamethasone, alternating with high doses of methotrexate and cytarabine) and CHOP (cyclophosphamide, doxorubicin, vincristine, and prednisone) or CHOEP (CHOP + etoposide), but some of these drugs are not available outside Japan [31]. Antiviral therapy with zidovudine (AZT) and interferon-α (IFN) has been frequently applied for ATLL in Europe and the USA, but the mechanism of action of AZT/IFN has been partially elucidated [32]. Therefore, studies about the safety and efficacy of new therapies, as well as existing ones, are essential to guarantee the treatment of patients. Our study evaluated articles produced in the last five years to verify the knowledge generated on this topic (Table 1).

Regarding the treatment schemes already existing, some studies have compared them and/or proposed alterations. A retrospective study of 103 untreated aggressive ATLL patients analyzed a modified EPOCH Regimen (mEPOCH; etoposide, vincristine, doxorubicin, carboplatin, and prednisolone). In the mEPOCH regimen, carboplatin was used as a substitute for the cyclophosphamide in order to avoid the drug efflux pump in ATLL cells due to the expression of P-glycoprotein, which leads to multidrug resistance. In these retrospective analyses, the overall response rate and complete response rate were 58% and 25%, respectively, with a median follow-up of 8.9 months; the median survival time was 9.8 months (95% confidence interval, 7.2–13.9 months). The median progression-free survival (PFS) was 4.2 months (95% confidence interval, 3.4–5.7 months). Patients who completed ≥4 cycles experienced significantly better overall survival and PFS compared with those who completed <4 cycles. Twenty-eight patients underwent allogeneic hematopoietic stem cell transplantation after mEPOCH and demonstrated significantly prolonged overall survival and PFS compared with those who did not. The authors concluded the mEPOCH was effective with tolerable adverse effects and prolonged overall survival mainly in patients that underwent allogeneic hematopoietic stem cell transplantation [33].

Regarding multiple drug regimens for the treatment of ATLL, there is the question of which combination would be the most effective and/or in which type of ATLL it should be used. A retrospective analysis of transplant-eligible patients with ATLL who received only VCAP-AMP-VECP or CHOP, analyzed by propensity scoring of inverse probability of treatment weighting (IPTW), investigated which regimen is a preferable front-line therapy in patients with aggressive ATLL in intermediate- and high-risk groups. The results showed that from 947 and 513 patients treated with VCAP-AMP-VECP and CHOP, respectively, the crude probabilities of two-year overall survival (OS) for patients in the VCAP-AMP-VECP and CHOP groups were 31.2% and 24.6%, respectively (*p* < 0.001). Stratified by risk group according to the modified ATL-prognostic index score at diagnosis, the crude probabilities of two-year OS in the VCAP-AMP-VECP and CHOP groups were, respectively, 39.8 and 45.0% in the low-risk group (*p* = 0.69), 32.2 and 21.6% in the intermediate-risk group (*p* = 0.001), and 17.2 and 6.2% in the high-risk group (*p* = 0.005). The authors suggested that the VCAP-AMP-VECP regimen is a preferable front-line therapy in patients with aggressive ATLL in intermediate- and high-risk groups, but it is important to emphasize that the trial was rather small, and no subsequent studies confirmed the benefit of VCAP-AMP-VECP over CHOP [34].

An Important target explored for the treatment of ATLL is the C-C chemokine receptor 4 (CCR4). Almost all patients (≥90%) with ATLL over-express this receptor and the mogamulizumab, a humanized defucosylated anti-CCR4 monoclonal antibody, has been studied for this purpose, being legally approved in Japan for the treatment of CCR4-positive ATLL [35]. In a prospective, randomized therapeutic trial that evaluated the efficacy and safety of mogamulizumab in ATLL patients with acute, lymphoma, and chronic subtypes with relapsed/refractory, aggressive disease in the US, Europe, and Latin America, the investigators did not find a result in terms of tumor response; however, mogamulizumab treatment resulted in an 11% confirmed overall response rate, with a tolerable safety profile (Table 2) [36]. The overall response found was compared with a previous phase II study of mogamulizumab monotherapy in 26 Japanese patients with relapsed CCR4+ ATL, which showed a 50% overall response rate (ORR), but the authors affirm several differences may account for this discrepancy. For example, the Japanese study included relapsed, not refractory patients, and confirmation of response was evaluated after 4 weeks (compared to 8 weeks in the author’s study) [37].

A study retrospectively analyzed 57 patients diagnosed with acute- and lymphoma-type ATLL in Japan. The results showed that the overall response rate to mogamulizumab was 54.2%. Median survival time (MST) and one-year overall survival (OS) rate from mogamulizumab initiation were 7.7 months and 42.0%, respectively. Patients with acute-type ATL showed longer MST (15.1 months) and higher one-year OS (63.6%). MST without skin rash was 5.0 months, and one-year OS was 34.3%; however, MST with skin rash was not reached, and one-year OS was 66.7%. Among patients who received the salvage therapy, longer MST and higher one-year OS were observed with mogamulizumab than without mogamulizumab (*p* = 0.078; 9.2 vs. 3.9 months; 47.9% vs. 17.6%, respectively). The findings suggested a significant anti-tumor effect of mogamulizumab against relapsed/refractory (r/r) ATLL [38].

However, the superiority of mogamulizumab over other salvage chemotherapies was not observed in general, with respect to overall survival (OS). It was only observed in patients with acute-type ATLL and skin rash. However, the assessment of skin adverse effects in clinical practice may be controversial, due to the subjective visual inspection of lesions [38].

The safety and effectiveness of mogamulizumab for the treatment of patients with (r/r) ATLL was also observed in another prospective, observational, post-marketing surveillance study with 572 patients, which concluded the best overall response rate and the response rate at the end of therapy were 57.9% and 42.0%, respectively. The median overall survival was 5.5 months, and survival was not different between patients aged ≥70 and <70 years, confirming it as a feasible option for the treatment of patients with r/r ATLL [39].

Drugs are also cited in the literature as useful for relieving symptoms and improving the quality of life of patients with ATLL. This is the case of etretinate, a synthetic retinoid analogue, which relieved the skin symptoms in cutaneous-type adult T-cell leukemia-lymphoma (cATL) at a daily dose of 10–40 mg in 9 cATL patients. The response of cutaneous lesions was evaluated by the modified response criteria for ATLL severity weighted assessment tools (mSWAT), before and 3 months after the introduction of the treatment. The remission was obtained in eight patients. Despite the adverse events observed in all patients but successfully controlled by the application of a moisturizer or a topical steroid, the limitations of the lack of controls, and the biases owing to the compliance or case selection, the authors suggest oral retinoids are a safe option for cATL patients, but studies with a larger sample population are needed (Table 2) [40].

Although clinical trials are extremely important to validate treatments, in vitro studies also play an essential role in the development of treatments. They help elucidate mechanisms of action and contribute to the search for new drugs or even the improvement of existing ones.

Among the drugs authorized for use by the US Food and Drug Administration and the European Medicines Agency, dimethyl fumarate (DMF) has been highlighted as a promising treatment for ATLL due to its effects in cancer cells, including cell signaling, proliferation, and cell death [41]. A study that examined the effects of DMF using the trypan blue exclusion assay and annexin V/propidium iodide staining in HTLV-1-infected and transformed T-cell lines (MT-1 and MT-2 cells), as well as evaluating its effects on the nuclear factor-kappa B (NF-ĸB), signal transducers, activators of transcription 3 (STAT3) signaling pathways, and anti-apoptotic proteins by immunoblotting, demonstrated the DMF inhibited proliferation and induced apoptosis in HTLV-1-infected and -transformed T-cells by suppressing NF-ĸB and STAT3 signaling pathways (Table 2) [42]. 

These results were observed by Maeta et al., and Sato et al., who evaluated the specific mechanism of DMF effects on the caspase recruitment domain family member 11 (CARD11)–BCL10 immune signaling adaptor (BCL10)–mucosa-associated lymphoid tissue lymphoma translocation protein 1 (MALT1) (CBM) complex and upstream signaling molecules that are critical for NF-ΚB signaling in MT-2 cells by immunoblotting. In the cited study, DMF inhibited proliferation and influenced the apoptosis of HTLV-1-infected cells by suppressing the CBM complex in the NF-ΚB pathway (Table 2) [43,44].

In the literature, apoptosis is highlighted as one of the main routes by which drugs act in the treatment of ATLL. In this context, we have dorsomorphin, an AMPK inhibitor that induced apoptosis in PBMCs from ATLL patients and in HTLV-1-infected T-cell lines in a dose- and time-dependent form. It increased the production of intracellular reactive oxygen species (ROS) that mediated DNA damage in HTLV-1-infected T-cell lines and suppressed the growth of human ATLL tumor xenografts in NOD/SCID mice [41]. 

A study with compounds belonging to the mesoionic class, synthesized under microwave irradiation, was evaluated against MT-2 and C92 cell lines infected with human HTLV-1, as well as in non-infected cell lines (Jurkat cells). These (*E*)-3-phenyl-5-(phenylamino)-2-styryl-1,3,4-thiadiazol-3-ium chloride derivatives were promising against cells, with IC_50_ values lower than 10 μM. The compounds with electron donor substituents (R=CH3 or OCH3) were more active, showing cell death by necrosis, interacting with DNA, and exhibiting lower hydrophobicity. Also, the preliminary pharmacokinetic profile obtained through human serum albumin (HSA) binding affinity using multiple spectroscopic techniques, zeta potential, and molecular docking calculations revealed that all the mesoionic compounds presented good binding parameters toward serum albumin, indicating feasible biodistribution in the human bloodstream (Table 2) [23].

Another important strategy to treat ATLL would be the use of antibody drug conjugates (ADCs) that have been recently introduced as part of anticancer therapy [45]. Yokota et al. constructed a novel ADC composed by an anti-CD70 single-chain Fv-Fc antibody conjugated with emtansine and evaluated it with regards to cell cytotoxicity and target specificity assessed by a cell proliferation assay. The results showed the anti-CD70 ADC selectively killed HTLV-1-infected cells and ATL cells without affecting other cells; however, additional studies with a larger number of peripheral blood mononuclear cells (PBMCs) from different patients are warranted for establishing this potential (Table 2) [45].

**Table 2:** New drugs, molecules, or natural products for adult T-cell leukemia/lymphoma treatment.

Adult T-Cell Leukemia/Lymphoma				
Drug/Molecule/Natural Product	Activity	Study Methodology	Year	Author
(*E*)-3-Phenyl-5-(phenylamino)-2-styryl-1,3,4-thiadiazol-3-ium chloride derivatives	The compounds showed cell death by necrosis and DNA interaction, especially those containing electron donor substituents.	Biological evaluation against MT-2 and C92 cell lines infected with human HTLV-1, and non-infected cell lines (Jurkat). Pharmacokinetic profile of the compounds was obtained through human serum albumin (HSA) binding affinity using multiple spectroscopic techniques (circular dichroism, steady-state and time-resolved fluorescence), zeta potential, and molecular docking calculations.	2020	[23]
Anti-CD70 single-chain Fv-Fc antibody conjugated with emtansine	Showed selective killing of peripheral blood mononuclear cells (PBMCs) from an ATLL patient.	Novel antibody drug conjugate (ADC) constructed using a novel antibody modification method. Its cell cytotoxicity and target specificity were assessed using a cell proliferation assay.	2020	[45]
Arsenic trioxide (As_2_O_3_)	As_2_O_3_ consolidation in combination with low-dose AZT/IFN maintenance may enhance long-term disease control in ATLL lymphoma with moderate side effects.	Retrospective study included nine newly diagnosed, previously untreated ATLL patients.	2020	[46]
Arsenic/interferon-alpha (As/IFN-α) with thymoquinone (TQ)	Led to a more pronounced and synergistic time-dependent inhibitory effect on HTLV-I-positive cells in comparison to As/IFN-α, as well as a significant decrease in tumor volume in a HuT-102 xenograft mouse model.	Trypan blue and flow cytometry were used to investigate viability and cell cycle effects. Annexin V staining, rhodamine assay, and Western blotting were used to determine apoptosis induction and changes in protein expression. Efficacy of single drugs and combinations were tested in an adult T-cell leukemia (HuT-102) mouse xenograft model.	2020	[47]
Dimethyl fumarate (DMF)	Inhibited proliferation and induced apoptosis in HTLV-1-infected and transformed T-cells by suppressing NF-ĸB and STAT3 signaling pathways.	Examined the proliferation and apoptosis by the trypan blue exclusion assay and annexin V/propidium iodide staining in HTLV-1-infected and transformed T-cell lines (MT-1 and MT-2 cells) and evaluated the NF-ĸB and STAT3 signaling pathways and anti-apoptotic proteins by immunoblotting.	2022	[43]
Dimethyl fumarate	Suppresses the proliferation of HTLV-1-infected T cells by inhibiting CBM-complex-triggered NF-ĸB signaling.	Assessed whether the BCL2 apoptosis regulator (BCL2)/BCL2-like 1 (BCL-xL) inhibitor navitoclax promoted the inhibitory effect of DMF on cell proliferation and apoptosis-associated proteins by trypan blue exclusion test and immunoblotting, respectively.	2023	[44]
Dorsomorphin	Induced apoptosis in PBMC from ATLL patients and dose- and time-dependent apoptosis in HTLV-1infected T-cell lines.	PBMCs were treated with dorsomorphin, stained with annexin V-fluorescein isothiocyanate (FITC) and 7-aminoactinomycin D (7-AAD), and analyzed by flow cytometry.	2020	[41]
Etretinate	It improves quality of life (QoL) by relieving the skin symptoms in cutaneous-type adult T-cell leukemia-lymphoma (cATLL).	Retrospective assessment of the efficacy and safety of etretinate in 9 patients with cATLL.	2019	[40]
Hypericin (HY) in photodynamic therapy (PDT)	It was highly effective against ATLL cells by induction of apoptosis and suppression of viral transcription.	Tested against ATLL cell lines and analyzed by colony formation assay, light and fluorescence microscopy, flow cytometry using an annexin V-FITC apoptosis detection kit, immunoblotting, luciferase assay, quantitative real-time PCR, chromatin immunoprecipitation assay, and HTLV-1 transmission assay.	2019	[48]
Modified EPOCH regimen	It was effective with tolerable adverse effects and prolonged overall survival mainly in patients that underwent allogeneic hematopoietic stem cell transplantation.	Retrospective analysis of untreated aggressive adult T-cell leukemia/lymphoma who received the modified EPOCH (mEPOCH) regimen.	2020	[33]
Mogamulizumab	The trial demonstrated the efficacy of mogamulizumab in comparison to other frequently used agents.	International, multicenter, open-label, randomized study conducted at 22 centers in Belgium, Brazil, France, Martinique, Peru, the UK, and the US; 18 centers screened and 17 randomized patients to determine the ORR of mogamulizumab that persisted and was confirmed at a subsequent response evaluation to compare cORR, PFS, OS, and DoR.	2019	[36]
Mogamulizumab	In clinical practice, mogamulizumab therapy was confirmed to be a feasible option for the treatment of patients with r/r ATLL, including the elderly, and the overall safety profile of mogamulizumab was manageable in most patients.	Prospective, observational, postmarketing surveillance conducted in patients with chemokine receptor 4 (CCR4)-positive, relapsed, or refractory adult T-cell leukemia-lymphoma (ATLL).	2019	[37]
Mogamulizumab	Exerts clinically meaningful antitumor activity in ATLL	Multicenter prospective observational study to establish the most effective and safe treatment strategy using mogamulizumab for ATLL patients.	2020	[39]
Mogamulizumab	Improved overall survival in patients with relapsed/refractory ATL, especially those with acute-type ATLL and skin rash.	Retrospective analysis of patients with acute- and lymphoma-type ATLL who received salvage therapy, and who received mogamulizumab	2020	[38]
Thymoquinone (TQ) with low concentrations of doxorubicin (dox)	Caused greater inhibition of cell viability and increased sub-G1 cells in both cell lines compared to dox or TQ alone. The combination induced apoptosis by increasing ROS and caused a disruption of mitochondrial membrane potential. TQ and dox combination also reduced tumor volume in mice more significantly than single treatments through enhanced apoptosis without affecting the survival of mice.	HTLV-1-positive (HuT-102) and HTLV-1-negative (Jurkat) CD4+ malignant T-cell lines were treated with TQ, dox, and combinations. Viability and cell cycle effects were determined by MTT assay and flow cytometry analysis, respectively. Combination effects on mitochondrial membrane potential and generation of ROS were assessed. Expression levels of key cell death proteins were investigated by Western blotting. A mouse xenograft model of ATLL in NOD/SCID was used for testing drug effects, and tumor tissues were stained for Ki67 and TUNEL.	2019	[49]
VCAP-AMP-VECP or CHOP	Significantly higher response rates and overall survival after treatment with VCAP-AMP-VECP than CHOP in transplant-eligible patients with aggressive ATLL.	Retrospective analysis of transplant-eligible patients with ATLL who received only VCAP-AMP-VECP or CHOP, incorporating inverse probability of treatment weighting (IPTW) using propensity scoring.	2019	[34]

ATLL: adult T-cell leukemia-lymphomas; AZT/IFN: zidovudine and interferon-alpha; BCL2: B-cell lymphoma 2; CHOP: combination cyclophosphamide-doxorubicin-vincristine-prednisone; cORR: confirmed overall response rate; DoR: duration of response; dox: doxorubicin nuclear; EPOCH: etoposide, prednisolone, vincristine, cyclophosphamide, and doxorubicin; LTR: long terminal repeats; modified EPOCH: etoposide, vincristine, doxorubicin, carboplatin, and prednisolone; NF-ĸB: factor-kappa B; OMDS: Osame motor disability score; ORR: overall response rate; PBMC: peripheral blood mononuclear cells; PDT: photodynamic therapy; PFS: progression-free survival; ROS: reactive oxygen species. STAT3: signal transducers and activators of transcription 3; TUNEL: deoxynucleotidyl transferase-mediated dUTP nick end labeling; VCAP-AMP-VECP-mLSG15: vincristine, cyclophosphamide, doxorubicin, and prednisone (VCAP), doxorubicin, ranimustine, and prednisone (AMP), and vindesine, etoposide, carboplatin, and prednisone (VECP).

Compounds from plants also have been studied for the treatment of ATLL. Hypericin (HY), a polycyclic quinone from *Hypericum perforatum* L., in photodynamic therapy (PDT), was tested against ATL cell lines. It was highly effective in inducing the inhibition of cell proliferation in an ATL cell with minimal effect on peripheral blood CD4+ T lymphocytes and caused apoptosis and G2/M phase cell cycle arrest in leukemic cells. Western blot analyses also revealed the downregulation of Bcl-2 and enhanced expression of Bad, cytochrome C, and AIF. The luciferase assay showed an enhanced expression of Bax and p53 proteins. Finally, the treatment suppressed the expression of viral protein HBZ and Tax by blocking the promoter activity via HTLV-1 5′LTR and 3′LTR, which highlighted the promising use of hypericin-PDT as therapy for ATLL [48].

Thymoquinone (TQ), obtained from *Nigella sativa* black seed, also was studied in combination with the anthracyclin doxorubicin (dox), whose major limitations in chemotherapy include tumor resistance and drug-induced severe side effects. TQ and dox caused greater inhibition of cell viability, induced apoptosis by increasing ROS and causing disruption of mitochondrial membrane potential, and reduced tumor volume in NOD/SCID mice more significantly than single treatments. Even though most of the mechanisms implicated in response to this combination model seem to be like those observed upon treatment with the drug alone, the authors suggest that this combination offers the possibility to use up to twofold lower doses of dox against ATLL with the same cancer inhibitory effects both in vitro and in vivo (Table 2) [49].

Recently, an innovative therapeutic perspective for the treatment of ATLL was discovered, paving the way for innovative research through viral biology. One study observed that the synergy between two transcription factors (IRF4/BATF3) makes ATLL viable, and when these factors are genetically knocked out, the cell cycle of these types of cancer is interrupted, as the MYC oncogene (target product of the activation of transcription factors’ transcription) is directly inhibited. Additionally, the study demonstrated that bromodomain-and-extra-terminal-domain (BET) chromatin protein inhibitors were able to act on the HBZ/BATF3 interface of cell lines from patients diagnosed with ATLL or xenotransplantation, performing cytotoxic action on all strains in in vitro preclinical tests [50].

**Figure 2:**
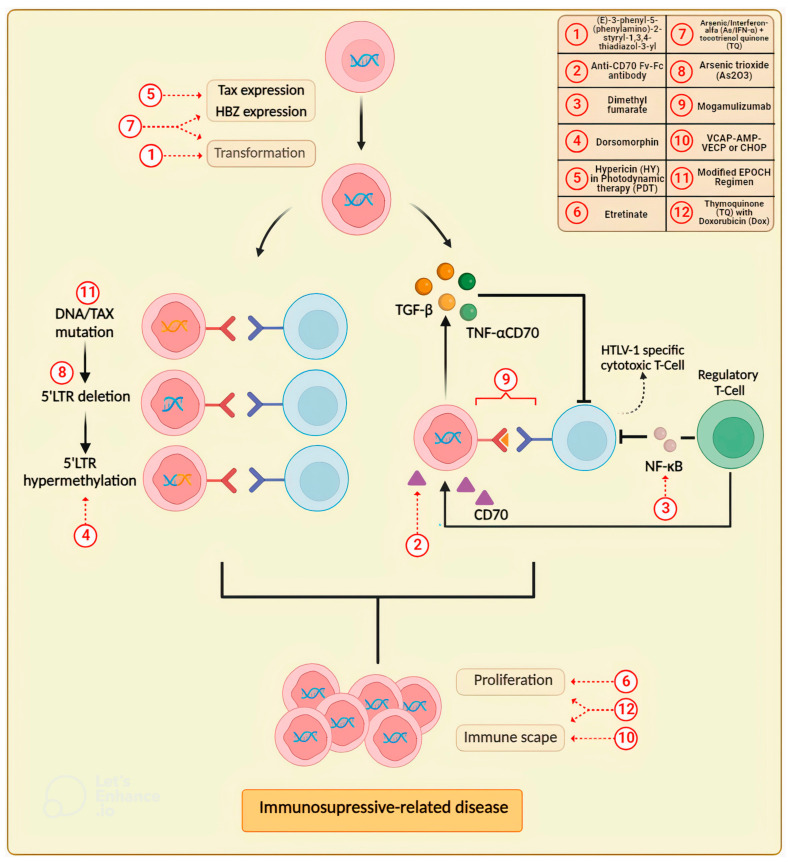
The pathological process of leukocyte mutation leading to the development of ATLL and potential sites of action of the studied molecules.

Below is an illustrative figure describing the pathological process of leukocyte mutation and the consequent damage leading to the development of ATLL, as well as the potential sites of action of the studied molecules. Figure 2 shows that the expression of Tax and HBZ in HTLV-1-infected cells results in continuous proliferation and inhibition of apoptosis, leading to cell immortalization. ATLL is associated with immunosuppression due to the accumulation of genetic alterations in infected T cells. Interactions with (*E*)-3-phenyl-5-(phenylamino)-2-styryl-1,3,4-thiadiazol-3-i lead to necrosis of infected cell lineages. The Fv-Fc anti-CD70 antibody induces the selective death of peripheral blood mononuclear cells in ATLL patients. Various therapies, such as dimethyl fumarate, dorsomorphin, etretinate, arsenic/interferon-alpha (As/IFN-α) with thymoquinone (TQ) [47], arsenic trioxide (As_2_O_3_) [46], and mogamulizumab, have specific actions to inhibit viral replication and induce apoptosis in HTLV-1-infected cells. Therapeutic plans VCAP-AMP-VECP or CHOP combine strategies to combat viral replication and immunosuppression. The modified EPOCH regimen and thymoquinone (TQ) with doxorubicin disrupt viral replication and induce apoptosis through free radical damage and the disruption of mitochondrial membrane potential in cells, respectively [51].

### 2.3. Drugs to Treat HTLV-1-Associated Myelopathy (HAM)/Tropical Spastic Paraparesis (TSP)

HTLV-1-associated myelopathy/tropical spastic paraparesis (HAM/TSP) is a progressive neuro-inflammatory disease [52]. HAM/TSP is characterized by spinal cord atrophy in the lower thoracic cord, perivascular demyelination, axonal degeneration, and inflammatory response caused by HTLV-1-infected CD4+ and CD8+ T cells [53]. This results in the destruction of nerve fibers, leading to a gradual loss of sensory-motor function. Symptoms include progressive muscle weakness in the lower limbs, stiffness, hyperreflexia, low back pain, paresthesia, and urinary and sexual problems [52,53,54].

Drug therapy for HAM/TSP caused by HTLV-1 cannot cure the disease. However, symptoms can be managed, and physiotherapy can contribute to patients’ functionality and quality of life, although the course of the disease is not altered. Currently, the use of corticosteroids, such as methylprednisolone in high doses and oral prednisolone (5 mg) for maintenance, is considered the best therapeutic option based on international consensus assessment and recommendations in the management of HAM/TSP to provide an evidence-based approach to the use of therapies made in accordance with the GRADE guidelines, a system for strength of evidence. According to this consensus, there is insufficient evidence to recommend interferon-alpha (IFN-α) as a first-line drug and antiretroviral therapy (treatment targeting HTLV-1 enzymes) for the treatment of HAM/TSP. It is important to emphasize that therapies to change the course of HAM/TSP must be applied to selected patients in order to maximize the benefits of treatment [19].

Although recommended, corticosteroid therapy has not yet been tested in a randomized clinical trial. Using this methodology, a phase 2 trial was performed to evaluate the effectiveness of corticosteroid therapy in patients with HAM/TSP. Patients were divided into two groups based on the speed of progression of motor impairments. In the fast-progressing group, those who received intravenous methylprednisolone plus oral prednisolone had one or more grade increase in the Osame motor impairment score, and 30% or more in the 10 m walk test from week 2 onwards. In the slow progression group, patients who received a single oral prednisolone treatment also showed a 15% improvement in the 10 m walk test, but only at the 24th week of the study [55].

In the last 5 years, some other drugs were studied as possible therapeutic options for HAM/TSP. Raltegravir, an antiretroviral that inhibits integrase, has been shown to successfully prevent in vitro transmission of HTLV-1 by interrupting the cell-to-cell contact necessary for the infection of healthy cells. Based on this result, a pilot, single-center, single-arm, open-label clinical study evaluated the effect of raltegravir on the proviral load and immune response of 16 patients with HAM/TSP. The patients received the antiretroviral drug twice a day for 6 months and were followed up for another 9 months without the drug. The results showed raltegravir did not equally reduce the proviral load in all patients. However, in the group in which there was a reduction in load, the expression of Tax and HBZ mRNA also decreased, with a reduction in Tax observed in the sixth month that was maintained until the fifteenth month of follow-up (Table 3) [56].

Schneidermann et al. (2022) sought to demonstrate in vitro the effects of antiretrovirals already used in therapy against the human immunodeficiency virus (HIV) as possible inhibitors for blocking HTLV-1 transmission. The study used a recombinant HTLV-1 integrase treated with increasing concentrations of cabotegravir, a recently developed drug that has been associated with efficacy in blocking HIV-1 transmission and replication, demonstrating similar results as raltegravir as an HTLV-1 integrase inhibitor. This study supports the possible use of antiretrovirals preventively in cases of exposure to HTLV-1 [57].

**Table 3:** New drugs for the HTLV-1-associated myelopathy/tropical spastic paraparesis treatment.

HAM/TSP				
Drug	Activity	Study Methodology	Year	Author
Intravenous methylprednisolone plus oral prednisolone	Increase of one or more grades in Osame motor disability score (OMDS) and improvement of 15% or more in the 10 m walking test.	Randomized, controlled phase 2 study. Patients were divided into rapid and slow progression of HAM/TSP. Patients with rapid progression were allocated into groups (1:1) to receive or not combined corticosteroid therapy. Patients with slow progression were allocated into groups (1:1) to receive oral mono corticosteroid therapy or not.	2022	[55]
Raltegravir	Unequal reduction in proviral load and expression of Tax and HBZ, with no changes in the evaluated scores.	A pilot, single-center, single-arm, open-label study treated 18 patients with 400 mg of raltegravir twice daily for 6 months. Patients were evaluated using the expanded disability status scale, scripps neurological rating scale, time 25 foot walk, Instituto Evandro Chagas scale, ambulatory index, and nine-hole peg test. Viral load and immunological markers were evaluated using blood and cerebrospinal fluid (CSF) samples.	2021	[56]
Teriflunomide	Dose-dependent regulatory action on the spontaneous proliferation of CD4^+^ and CD8^+^ T lymphocytes; however, CD25 continued to be expressed, as well as the expression of Tax and HBZ mRNA and Tax protein.	PBMCs from 12 patients with HAM/TSP were collected and cultured in the presence and absence of teriflunomide to test cell viability, lymphocyte proliferation, activation markers, and the expression of Tax and HBZ mRNA and Tax protein.	2021	[58]
L-arginine	Increase in walking speed in the 10 m walk test over 14 days and improvement in gait function in the timed Up and Go test on the 14th and 28th days, along with a reduction in neopterin levels.	Phase 2, open-label, single-arm study: 20 HAM/TSP patients received oral L-arginine (20 g) for 1 week, followed by 3 weeks of observation without treatment. Assessments included walking tests, evaluation of inflammatory markers, and safety/tolerability.	2023	[59]
Prosultiamine	There was a decrease in night-time frequency, urgency, and levels of biomarkers for overactive bladder, such as nerve growth factor/creatinine and adenosine triphosphate/creatinine.	Prospective, single-center, open-label study. The patients received a once-daily treatment of 300 mg of prosultiamine, and their symptoms of overactive bladder and biomarkers were assessed at baseline and after 12 weeks.	2019	[60]

Teriflunomide, a dihydrooratate dehydrogenase inhibitor, was tested on samples from 12 patients with HAM/TSP caused by HTLV-1. Patients were not using corticosteroids and had been diagnosed with HTLV-1 for over 10 years. The collected cells were cultured and subjected to tests to assess lymphocyte proliferation; expression of activation markers, Tax, and HBZ mRNA; and Tax protein expression. The results showed that teriflunomide exerted a dose-dependent regulatory action on the spontaneous proliferation of lymphocytes. The best response was observed at the 100 μM dose, resulting in a 90.7% inhibition, preventing the abnormal proliferation of CD4+ and CD8+ T lymphocytes, although markers of lymphocyte activation, such as CD25, were still expressed. There was no change in Tax and HBZ mRNA expression, as well as in Tax protein expression (Table 3) [58].

A phase 2, open-label, single-arm clinical trial was conducted with 20 patients diagnosed with HAM/TSP to evaluate the efficacy and safety of short-term treatment with L-arginine. During the study, patients received 20 g of L-arginine three times a day for one week; then, the amino acid was discontinued, and patients were followed for 3 weeks, evaluating them by the 10 m walk test, timed Up and Go test (TUGT), and neopterin levels produced after stimulation via interferon-gamma (IFN-γ). Patients demonstrated an increase in walking speed on the 10 m walk test on day 14 and improvement in gait functions on the TUGT on days 14 and 28, in addition to a reduction in neopterin levels in the cerebrospinal fluid of 12.7 pmol/mL to 10.6 pmol/mL. Adverse effects were minimal (Table 3) [61].

HAM/TSP also causes urinary disorders, such as an overactive bladder, and affects patients’ quality of life. In the search for alternatives to treat these symptoms, a prospective, single-center, open-label study evaluated the 12 week use of 300 mg of prosultiamine, a homologue of allithiamine, in 16 patients with HAM/TSP-related overactive bladder, evaluating nocturnal urination frequency, urinary urgency, and nerve growth factor/creatinine and adenosine. There was a decrease in biomarker concentrations and less nighttime urination and urinary urgency, with no reports of relevant adverse effects during the trial [62].

**Figure 3:**
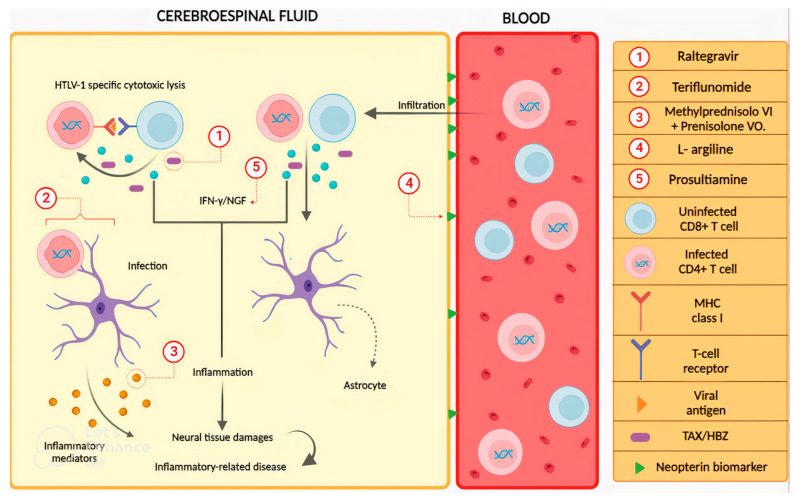
Pathological process associated with HAM/TSP and possible sites of action of the studied molecules. Adapted from Futsch, Mahieux, Dutartre et al., 2017 [51].

Below is an illustrative figure depicting the pathological process of leukocyte infiltration and the consequent injury that leads to the development of clinical manifestations associated with HAM/TSP, as well as the possible sites of action of the studied molecules. The HAM/TSP is related to the production of IFN-γ (interferon-gamma) and NFG (nerve growth factor) by HTLV-1-infected cells and HTLV-1-specific cytotoxic cells infiltrating the cerebrospinal fluid, leading to damage to neural tissues, both directly through the exacerbation of inflammatory responses and indirectly by recruiting more IFN-γ-producing cells into the cerebrospinal fluid. Thus, the action of antiretrovirals raltegravir [56] and teriflunomide [58] is focused on reducing the expression of the proteins Tax and HBZ [46], which are essential for viral replication and regulating the expression of new CD4+ and CD8+ T cells. On the other hand, the intravenous methylprednisolone plus prednisolone oral treatment showed a reduction in the rate of pro-inflammatory cytokines produced by astrocytes [55]. Finally, prosultiamine demonstrated improvement in the inflammatory response by reducing the expression of IFN-γ and NFG [62], a mechanism different from the one proposed by L-arginine supplementation, which possibly shows a reduction in leukocyte infiltration through the reduction in neopterin levels [61] (Figure 3).

### 2.4. Challenges and Prospects in the Drug Treatment of HTLV-1-Associated Strongyloidiasis

Strongyloidiasis is a neglected cosmopolitan disease worldwide, endemic in tropical and subtropical regions with an estimated 100 million infected individuals. Infection in humans occurs mainly by *Strongyloides stercoralis*, a nematode of the Strongyloididae family that causes chronic and asymptomatic infections [59,60,63]. *S. stercoralis* can lead to the development of hyperinfection and disseminated infection, especially in immunosuppressed individuals such as HTLV-1 carriers. The coinfection of *S. stercoralis* and HTLV-1 is well established in the literature, with the description that the two pathogens share the same endemic areas. Furthermore, patients with HTLV-1 often develop the disseminated form of strongyloidiasis, as the co-infection favor the clonal proliferation of cells infected by HTLV-1, as well as the development of *S. stercoralis* larvae in the host [59,60,63].

**Figure 4:**
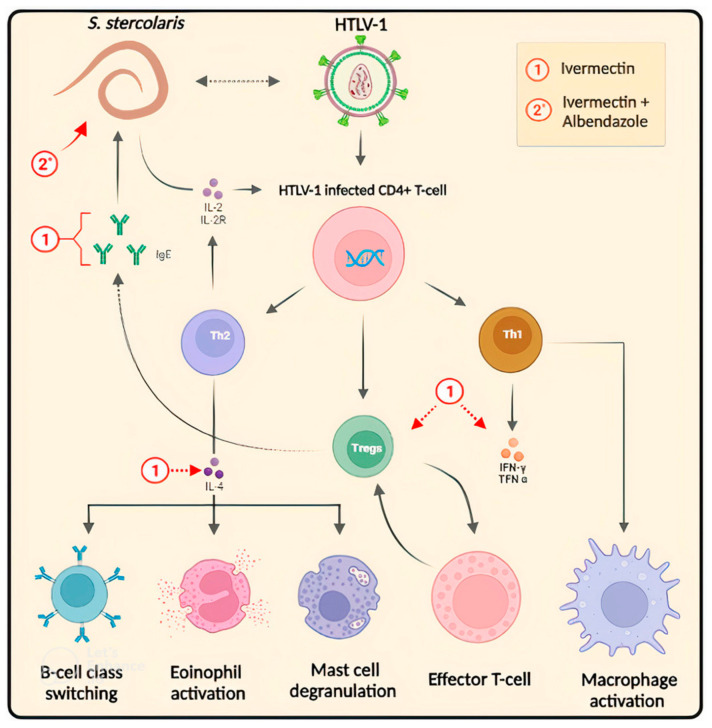
Mechanism HTLV-1/*S. stercoralis* co-infection and drugs interference target during infection process.

In the mechanism of HTLV-1/*S. stercoralis* co-infection, CD4^+^ T cells infected with HTLV-1 induce a predominant Th1 response, elevating IFN-γ and TNF-α production. This reduces IL-4, inhibiting B cell isotype switching to IgE and decreasing eosinophil and mast cell activation. The increased Th1 response also dampens Th2 response with IL-10 release. HTLV-1 is associated with an upregulation of Treg cells (CD4^+^CD25^+^FOXP3^+^), which inhibit effector T cells and decrease Th2 response observed in the co-infection. The reduced IgE production and diminished activation of eosinophils and mast cells hinder the elimination of *S. stercoralis*, resulting in disseminated strongyloidiasis. Additionally, *S. stercoralis* dissemination induces IL-2/IL-2R, leading to polyclonal expansion of HTLV-1-infected T cells and sequential events. Thus, the multifactorial action of ivermectin treatment is highlighted, regulating cytokines and the immune response of Tregs, IFN-γ, TNF-α, IL-4, and IgE, in contrast to albendazole treatment, which acts directly on the worm [59,60] (Figure 4).

The hyperinfection syndrome (HS) and disseminated strongyloidiasis in a co-infection with *S. stercoralis* and HTLV-1 can be fatal if not treated. Several studies have demonstrated the efficiency of treating this disease with ivermectin, achieving a complete cure in most cases. However, this success has been related to other factors, such as the correct and early diagnosis of coinfection, with attention to signs and symptoms [59,60,63].

When considering the impact of HTLV-1 on anti-*S. stercoralis* treatment response, a systematic review and meta-analysis conducted by Ye et al. (2022) evaluated 14 articles and concluded that people living with HTLV-1 have a higher risk of being infected by *Strongyloides* and have a higher risk of developing severe strongyloidiasis and anthelmintic treatment failure. The effectiveness of treatments was determined by stool examinations with a post-treatment follow-up duration ranging from 3 to 12 months. According to the authors, regardless of treatment type (6 mg ivermectin, repeated after 2 weeks; 200 μg/kg ivermectin, repeated after 2 weeks; 400 mg/day albendazole for 3 days, repeated after 2 weeks; 5 mg/kg pyrvinium pamoate for 3 days, repeated after 2 weeks), HTLV-1 co-infected patients had higher odds of having failed antiparasitic therapy than those without HTLV-1 [64]. The most widely used and most effective agent was ivermectin, but Hoces et al. reported that all patients that had been treated with higher dosages of ivermectin showed negative results after treatment. However, late recurrences, even after two courses of appropriate doses of ivermectin, may occur months or years later. In HTLV-1 co-infection, prolonged surveillance post-*Strongyloides* treatment is essential [59].

A previous study by Gordon et al. (2020) corroborates these findings and adds that the same can also be observed for drugs such as albendazole and thiabendazole, discussing the greater probability of failure or reduced effectiveness in individuals with HTLV-1 infection, demonstrating the resistance of *S. stercoralis* in the presence of the virus (Table 4) [60].

An important issue regarding treatment is the access to ivermectin. In many strongyloidiasis-endemic countries, this drug is restricted, and generic prequalified ivermectin is not widely available. The situation is further compounded by the future prospect of *S. stercoralis* resistance due to repeated mass drug administrations as part of control programs for onchocerciasis or lymphatic filariasis elimination. Thus, the development of alternative treatment options to ivermectin that serve as additions to the depleted drug armamentarium are urgently needed [65].

**Table 4:** Drugs used in the treatment of HTLV-1-associated strongyloidiasis.

Strongyloidiasis				
Drug	Activity	Study Methodology	Year	Author
Ivermectin 200 mcg/kg	In addition to the risk of developing multiorgan failure syndrome, a fact observed in the patient after starting ivermectin, following the neurological evolution, was unfavorable.	A case report illustrating the risk of occurrence of bacterial infections such as Gram-negative meningitis in the case of disseminated infection in a patient co-infected by Strongyloides-HTLV-1.	2021	[65]
Ivermectin 200 mcg/kg, albendazole 400 mg	In the patient, the medical procedure was initiated with broad-spectrum antibiotic therapy for bacterial co-infection, but despite this, there was no clinical response of improvement, leading to a fatal outcome.	A case report describing the clinical case of a patient with a history of HTLV-1 infection and ulcerative colitis that developed into *Strongyloides stercoralis* hyperinfection, including its diagnosis and treatment.	2021	[66]
Ivermectin 200 μg/kg	Treating strongyloidiasis infection decreases circulating Tregs, but antigen-specific cytokine remains altered. This may reflect the blunting of sensitization by Tregs.	Diagnosis of strongyloidiasis was made by stool examination using Baermann’s sedimentation method. All patients positive for Strongyoides larvae were tested for HTLV-1 infection by ELISA. Positive results were confirmed by Western blot. Then, patients received antihelminthic treatment with ivermectin 200 μg/kg dose on two consecutive days. A second course of two doses was given 15 days later. Clinical follow-up included the assessment of treatment efficacy by Baermann’s exam on stool. Then, flow cytometry and antigen-specific cytokine response tests were performed.	2020	[59]
Subcutaneous ivermectin, albendazole	Subcutaneous ivermectin was used as an anthelmintic treatment with an adequate therapeutic response.	A case report described a case of a man coinfected with *Strongyloides stercoralis* and HTLV-1.	2019	[67]

The authors Guérin et al. (2021) and Rivera et al. (2021) discussed the clinical case of two patients who were hospitalized due to diarrhea, vomiting, and fever, one of whom had neurological impairment. The two patients rapidly worsened, and only one of them had a previous history of HTLV-1 infection, being treated for HAM/TSP. In the cases described, the diagnosis for strongyloidiasis and/or HTLV-1 did not occur right at the beginning of the investigation, and the patients presented other conditions, such as bacterial meningitis and ulcerative colitis. Both cases were treated with ivermectin 200 mcg/kg, and only one patient received auxiliary treatment with albendazole 400 mg per day. Unfortunately, in both cases, the outcome was death, maybe due to disseminated infection and late diagnosis, highlighting that patients with HTLV-1 are more likely to develop severe strongyloidiasis and failure in treatment with anthelmintic drugs (Table 4) [65,66].

Hunter et al. (2019) reports the clinical case of a man residing in an Argentine province who reported diarrhea, abdominal pain, and weight loss in recent months. This case was treated as a severe inflammatory bowel disease; however, imaging tests and biopsy revealed the presence of *S. stercoralis* larvae. In view of this, the patient was tested for HTLV-1, demonstrating the co-infection. Then, he was treated with subcutaneous ivermectin and albendazole due to the extensive involvement of the intestine, which prevents good drug absorption. Gradually, an improvement in the clinical condition was observed, proving that subcutaneous administration could be an option in similar cases [67].

Hoces et al. (2020) analyzed the hypothesis that the ivermectin could act on the expansion of regulatory T cells (Tregs) in contribution to the restoration of the cytokine response, improving the antigen-specific response. The study analyzed twenty asymptomatic people, but with active infection by *S. stercoralis*, in which six were cases of co-infection with HTLV-1. The participants received treatment with ivermectin of 200 mcg/kg over 2 consecutive days, and 15 days later, the effectiveness of the treatment was assessed through the Bearmann technique in the stool, confirmed by PCR, and the response of cytokines was verified through flow cytometry. The study demonstrated Tregs are indeed influenced by *S. stercoralis*, especially in co-infected patients; therefore, an effective treatment for strongyloidiasis can confer benefits by inhibiting the parasite’s synergistic effect on the expansion of Tregs and promoting better immune responses in HTLV-1-positive patients (Table 4). Further studies on this topic may serve as a basis for discovery of new drugs that act on the expansion of regulatory Tregs for treatment of HS and disseminated strongyloidiasis in patients co-infected with *S. stercoralis* and HTLV-1 as an alternative to ivermectin [59].

Finally, Dykie et al. (2020) argue that a relevant point to find new drugs to strongyloidiasis is the need to develop new animal models with a more appropriate size to allow for detailed studies of how the interaction between these two pathogens occurs and what drugs may interfere with co-infection and spread of the parasites [68].

### 2.5. Molecular Docking as a Tool to Discover New Drugs against HTLV-1 Infection and Related Diseases

A method prominent in recent studies for drug discovery is molecular docking (MD). This technique is used to predict the preferred position of a binding molecule in a receptor macromolecule and evaluate physical-chemical properties related to their biological activity [69]. MD can identify new drugs for the treatment of HTLV-1 through the prediction of interactions between small molecules and essential proteins in the targeting of viral enzymes and host factors involved in both HTLV-1 replication and problems associated with infection [70].

Studies have described the progression of HTLV-1 infection, inflammatory conditions, and the occurrence and severity of related diseases as consequences of HTLV-1 replication in CD4+ T and CD8+ T lymphocytes, as well as the imbalance between proinflammatory and anti-inflammatory cytokines [59,71]. There are several protein targets in HTLV-1, such as protease (PR), reverse transcriptase (RT), and integrase (IN), which are ultimately involved in viral replication and retroviral pathogenesis [72]. For example, HTLV-1 protease is an aspartic protease crucial for the maturation and replication cycle of HTLV-1, with 28% similarity to HIV-1 protease and 45% identity between active site residues. The blockage of HIV-1 PR enzyme has shown to be reliable in battling the virus [72]. Additionally, HTLV-1 PR is important for viral growth and replication by cleaving the viral Gag and Gag-(Pro)-Pol polyproteins and aiding the maturation of structural and functional proteins of the virus [73]. Consequently, hindering HTLV-1 PR can be a reliable treatment against HTLV-1 infections, demonstrating that some target proteins of anti-HIV treatment may be promising for anti-HTLV-1 treatment [74]. 

In addition, the cost of drug discovery and development can reach up to 2.6 billion dollars [75], but by using drug repurposing, the cost can be drastically reduced because it is a process of finding new indications for already tested and approved drugs, which can be very efficient and timesaving, as all the safety tests have been done and only the efficacy of the potential drug must be tested in clinical trials. Even the design of new antiretrovirals more specifically focused on HTLV-1 targets based on existing antiretrovirals can save money and time. Computational tools and methods such as molecular docking and molecular dynamics (MD) simulation are widely used methods in the field of drug discovery and drug design because they enable the study of 3D structures and interactions of protein–ligand complexes in great detail with the possibility of finding potential binders [70]. 

The recent study by Jahantigh et al. (2022) used molecular docking to identify potential protease inhibitors among approved antiviral drugs as a potential treatment against HTLV-1. In general, protease is an essential enzyme for the viral replication cycle and therefore represents a promising target for the development of antiviral therapies. In the study, molecular docking techniques and molecular dynamics simulation were used to evaluate the interaction of different antiviral drugs with HTLV-1 protease [73]. The results showed that some antiviral drugs (mainly simeprevir, atazanavir, and saquinavir), originally developed to fight other viruses, presented a significant affinity with the HTLV-1 protease. This finding suggests the possibility of repurposing these drugs for the treatment of HTLV-1 infection that could speed up the process by leveraging already available knowledge and resources (Table 5) [73].

To take advantage of knowledge about existing antivirals and reconstruct the binding pathway of an anti-HIV drug indinavir, in a complex with HTLV-1 protease, Sohraby and Hassan (2021) used molecular dynamics simulations. Indinavir is a protease inhibitor, and the simulation made it possible to follow the molecular pathway of indinavir binding involved in this process. The reconstruction of the binding pathway of indinavir with the HTLV-1 protease provided valuable information on the molecular mechanisms involved in this interaction for developing new treatment solutions against HTLV-1 [76].

**Table 5:** Molecules and targets analyzed in the articles found in the databases.

Molecular Doking				
Molecule	Target	Docking Methodology	New Findings	Author
Simeprevir, atazanavir, and saquinavir	Key residues in the HTLV-1 protease binding site.	Molecular simulation between drug, target protein, and the interaction between them.	It suggests the possibility of repurposing these drugs for the treatment of HTLV-1 infection.	[73]
Multiepitope immunization	Multiepitope domain targeting viral antigenic regions.	Molecular dynamics between the immunizer and the immune system.	The engineered vaccine has shown promise in structural stability and ability to induce an immune response.	[77]
Chloride derivatives (5a–d)	Sites of HTLV-1-infected MT-2 and C92 cell lines.	Simulations to predict how these compounds bind and interact with the replication target protein.	HSA-5 compounds can be active in HTLV-infected MT-2 and C92 cells, with potential bioavailability.	[23]
Indinavir	HTLV-1 protease reactive sites.	Conformational interaction between indinavir and HTLV-1 protease at an atomic level.	Simulation of indinavir binding to protease, revealing its molecular interactions.	[76]
Modified naphthyridines	Deltaretroviral intasome domains.	Analysis of structures of deltaretroviral intasomes obtained by cryo-EM.	Existence of specific interactions between intasome components and proviral DNA.	[78]

Barski et al. (2020) analyzed a structural basis for the inhibition of HTLV-1 integration through the analysis of deltaretroviral intasome structures obtained by electron cryomicroscopy (cryo-EM). HTLV-1 integration is an essential process for viral replication, and understanding the molecular details involved in this process may lead to the development of more effective therapeutic strategies against HTLV-1. Analysis of the structures revealed specific interactions between intasome components and viral DNA, providing insights into the mechanisms of DNA recognition and binding during the integration process. Furthermore, the researchers identified critical regions of the intasome that could be targets for the development of specific inhibitors [78].

Sousa-Pereira et al. (2020) also used MD to obtain theoretical results to evaluate the activity of chloride derivatives, synthetic compounds that are promising chemotherapy agents in HTLV-1 infection. Their study showed some of the thiadiazole chloride derivatives demonstrated potent activity against HTLV-1, suggesting that the mechanism of action of the most promising compound could be related to its capacity to intercalate into DNA [23]. 

An interesting alternative against viruses is vaccines. The study carried out by Alam et al. (2020) explored all the possible strains of HTLV-1 using different data-driven tools, including immunoinformatics, to predict an efficient vaccine. Using Vaxijen, which predicts antigens based on the biophysical and physicochemical properties of proteins, as well as other tools, the authors identified an envelope glycoprotein, GP62, of HTLV-1 as a probable antigen. It was targeted for designing the vaccine because glycoproteins are located on the outer layer of the cell membrane and can easily be recognized by the immune system. It also fits into parameters such as the Shannon entropy (H), which was selected as a variability metric, and the variability threshold was set at 0.5. Afterward, only the conserved epitopes were picked, and they coincided entirely through their whole length. After conducting a rigorous analysis with numerous databases and using tools of docking methods, nine epitopes for MHC (major histocompatibility complex) class I and five epitopes for MHC class II were selected. Among all the predicted peptides, the epitopes ALQTGITLV and VPSSSTPL interacted with three MHC alleles, and the author estimated that worldwide coverage for these alleles was about 70%. Docking revealed that ALQTGITLV and VPSSSTPL epitopes interact strongly with the epitope-binding groove of HLA-A02:03 and HLA-B35:01, respectively, and that molecular dynamics simulations of the bound complexes revealed that they form stable complexes. Therefore, this study revealed promising options for the development of a vaccine against HTLV-1 [79].

In the same sense, Jahanting et al. (2021) used bioinformatics programs to investigate possible candidates for producing immunobiologicals against HTLV-1. Investigating five proteins (Hbz, Tax, Pol, Gag, and Env), the researchers identified a strong candidate with a domain of eight epitopes highlighted both in B cells and in T cells. To verify the possible interactions between the epitopes of the projected protein and immune cell receptors, in silico docking studies were conducted, demonstrating strong interaction and the formation of complexes O2–HLA-A02:01 and D8–HLA-A02:01, which remained stable throughout the simulation. The results demonstrated an interaction between the projected protein and B- and T-cell receptors, producing humoral and cellular immune responses [80].

Another interesting perspective was tested by Pandey et al. (2019), which used computer simulations to present a combinatorial screening algorithm to design a multiepitope vaccine targeting HTLV-1 infection. The authors combined different amino acid sequences to form a multiepitope vaccine and subjected it to analyses and computer simulations to assess its structural stability and its ability to interact with the immune system. They concluded this vaccine can induce an effective immune response against HTLV-1, targeting multiple antigenic regions of the virus and increasing the likelihood of protection against infection [77].

## 3. Conclusions and Future Prospects

The human T-lymphotropic virus type 1, HTLV-1, is a high-impact viral infectious agent whose mechanisms of pathogenesis are not completely defined. Furthermore, the existence of diseases related to the infection, such as HAM/TSP, strongyloidiasis, and ATLL, of varying courses and severities, make treatment against the infection a challenge for the scientific community. There is an international consensus regarding the treatment of ATLL and HAM/TSP to define prognostic factors, clinical subclassifications, treatment strategies, and response criteria. It uses GRADE criteria for quality of evidence and affirms that for ATLL, the level of evidence should be regarded as low or very low—the equivalent of a GRADE evidence score of C or D. For HAM/TSP, there is evidence to support the use of both methylprednisolone and prednisolone as for progressive disease, but there is no evidence to support the use of other therapies including antiretrovirals and interferon-α. Therefore, although the consensus exists, it does not determine the standardized use of drugs as the first line of treatment, because not all drugs are available and approved for use in countries with the highest prevalence of HTLV-1 infection.

However, the observation of existing literature as well as the use of molecular techniques and in vitro and in vivo studies can help to keep the consensus updated and, in the future, help in the elaboration of guidelines with alternative treatments and/or more effective drugs. In our review, the category with the highest number of articles was “drugs for the treatment of ATLL”, perhaps due to the severity associated with this disease or the fact that the virus was first isolated from a person with a cutaneous T-cell malignancy. 

Another interesting thing was the fact that compounds from very different classes and origins were studied and showed promise for the treatment of ATLL. Among the selected articles, we observed compounds from various classes, ranging from mesoionic compounds to antineoplastics such as arsenic trioxide, as well as antibody drug conjugates, and moreover natural products such as thymoquinone and hypericin. This demonstrates that many classes of compounds can be used to treat ATLL.

Articles regarding the treatment of HAM/TSP also demonstrated a wide variety of classes, including immunomodulators; antiretrovirals; and even arginine, which is an amino acid that seems promising in the palliative treatment of the inflammatory process, thus increasing the patient’s quality of life.

Regarding strongyloidiasis, only a few articles on this topic were included, with most of them being case reports. In these reports, the authors highlighted that co-infection may be a determining factor in the difficulty of responding to treatment with ivermectin, which is considered the first choice for strongyloidiasis. Additionally, the negative prognosis has been attributed to late diagnosis or association with HTLV-1, suggesting that this factor could directly influence treatment failure.

Further studies are needed to assess the effectiveness of these compounds, including the use of molecular docking and computer simulations to discover new therapeutic targets, design vaccines, or even repurpose existing antiviral drugs. 

The weaknesses of this study may reside in the few controlled and randomized clinical trials in the last 5 years, which may make it difficult to obtain conclusive evidence on the efficacy of the drugs under analysis. In addition, the heterogeneity of the included studies can make comparison and generalization difficult, due to differences in patient populations, diagnostic criteria, treatment protocols, and evaluated outcomes, as well as the intrinsic limitations of primary studies, such as small samples, lack of adequate control of variables, and insufficient duration of follow-up.

This study provides a careful and comprehensive review of the most up-to-date evidence on new drugs and therapies under development, identifying opportunities for developing more effective and targeted treatments, as well as promoting the visualization of gaps in knowledge and providing guidance for future research and clinical trials because it systematized promising therapeutic targets and potential treatments for various conditions associated with HTLV-1. Through data synthesis, this document is expected to provide better insights for the scientific community, professionals, and health managers regarding the latest research on drugs against HTLV-1 infection and related diseases, supporting proposals for treatment. 

## Data Availability

Data sharing is not applicable.
